# Higher dietary intakes of choline and betaine are associated with a lower risk of primary liver cancer: a case-control study

**DOI:** 10.1038/s41598-017-00773-w

**Published:** 2017-04-06

**Authors:** Rui-fen Zhou, Xiao-Lin Chen, Zhong-guo Zhou, Yao-jun Zhang, Qiu-ye Lan, Gong-cheng Liao, Yu-ming Chen, Hui-lian Zhu

**Affiliations:** 1 School of Public Health, Sun Yat-sen University, Guangzhou, 510080 P.R. China; 2 Department of Hepatobiliary Surgery, Sun Yat-sen University Cancer Center, Guangzhou, 510080 P.R. China

## Abstract

The dietary intake of methyl donors is favorably associated with many diseases, but the findings regarding primary liver cancer (PLC) risk are limited. This study investigated the association between the intake of choline, betaine and methionine and PLC risk in adults. This 1:1 matched case-control study enrolled 644 hospital-based PLC patients and 644 community-based controls who were matched by sex and age, in Guangzhou, China. An interviewer-administered questionnaire and a food-frequency questionnaire were used to collect general information and dietary intake information. Conditional logistic regression showed a significantly inverse association between total choline and betaine intakes and PLC risk. The multivariable-adjusted odds ratios (ORs) and their 95% confidence intervals (CIs) for PLC for the top (*vs*. bottom) tertile were 0.34 (0.24–0.49; *P*_*-*trend_ < 0.001) for total choline and 0.67 (0.48–0.93; *P*_*-*trend_ = 0.011) for betaine. No significant association was observed between the intake of methionine and PLC risk (*P* > 0.05). For individual choline compounds, higher consumptions of free choline, glycerophosphocholine, phosphocholine, phosphatidylcholine and sphingomyelin were associated with a lower PLC risk (all *P*-trend < 0.05). The studied associations were not significantly modified by the folate intake (*P*-interactions: 0.488–0.890). Our findings suggest that higher choline and betaine intakes may be associated with a lower risk of PLC.

## Introduction

Primary liver cancer (PLC), one of the most highly malignant tumors with a poor prognosis, is the sixth most common cancer and the third leading cause of cancer-related mortality worldwide^1^. Additionally, half of the cases and deaths are estimated to occur in China^1^. Previous studies have shown that persistent infection with hepatitis B or C virus (HBV/HCV) is the leading cause of PLC^2^. Other established risk factors include aflatoxin-contaminated foods, excessive alcohol consumption, fatty liver, metabolic syndrome, and tobacco smoking^3, 4^. However, many studies have shown that dietary factors may also play an important role in the development of PLC^5–7^.

Choline, which is an essential nutrient, may protect against PLC through its effect on the one-carbon metabolism, structural integrity and signaling functions of cell membranes^8^. Choline can be irreversibly oxidized to betaine, providing one-carbon units in the conversion of homocysteine (Hcy) to methionine and the generation of the universal methyl donor S-adenosylmethionine (SAM)^9^. Previous studies have shown that one-carbon metabolism involves alterations in the biosynthesis of nucleotides and DNA methylation, which are thought to be implicated in carcinogenesis^10, 11^. Thus, it was hypothesized that the dietary intake of one-carbon related nutrients, such as choline, betaine and methionine, may play an important role in the development and progression of cancer.

Several prospective and retrospective studies have addressed choline, betaine and methionine intake in relation to cancer risk. For breast, colorectal and lung cancer, associations have been inverse^12–17^ or null^18, 19^. However, for colorectal adenoma^20^ and lethal prostate cancer^21^, an adverse association for choline has been reported. Reduced risks at higher intakes have also been reported for renal cell^22^, nasopharyngeal^23^ and pancreatic cancers^24^. However, for gynecological malignancies^19, 25^, associations have consistently been null. A recent meta-analysis showed that an increase of 100 mg/day of choline plus betaine intake might reduce the cancer incidence by 11% (0.89, 95% CI, 0.87 to 0.92)^26^. To the best of our knowledge, however, only one case-control study has examined the associations between the serum concentrations of betaine, choline, methionine and risk of hepatocellular carcinoma in the Chinese population in Shanghai^27^. No study has yet examined the associations between the dietary intake of choline, betaine and methionine and risk of PLC in humans. To address this issue, we investigated the associations between the intake of choline, betaine, and methionine and risk of PLC in a Chinese population.

## Materials and Methods

### Study population

From September 2013 to January 2016, a 1:1 matched case-control study was conducted in Guangzhou, China. One thousand forty PLC cases aged 18–80 years were recruited from the Sun Yat-Sen University Cancer Center. All PLC cases were newly diagnosed within one month and were confirmed using tissue evaluation or liver Magnetic Resonance Imaging (MRI) according to the National Comprehensive Cancer Network (NCCN) *Clinical Practice Guidelines in Oncology: Hepatobiliary Cancers* ^28^. We excluded PLC patients if they had any of the following conditions: (1) history of PLC or any other cancer (49 cases); (2) chronic diseases, such as hospital-confirmed diabetes, stroke, myocardial infarction or heart failure, kidney failure, chronic liver diseases, physical or mental disabled, or other diseases/conditions that may have changed their dietary habits or affected their routine activities within the previous 5 years (122 cases); (3) refusal to participate or incompletion of the survey (215 cases). We screened all inpatients with a PLC diagnosis indicated in the first page of the case report form for their eligibility during the study period. We further excluded those who completed the dietary survey with an implausible total energy intake (<500 or >3500 kcal per day for women and <700 or >4200 kcal per day for men) (10 cases). A total of 644 (559 men, 85 women) eligible patients were included in the analysis. Among the case patients, 292 (45.3%) were diagnosed by tissue biopsy, and 352 (54.7%) were diagnosed by MRI imaging. Of those with tissue evaluation, 272 (93.2%) were hepatocellular carcinoma (HCC) (both ICD-9 code 155.0 and ICD-O-2 Morphology code 8170/3), 16 (5.5%) were cholangiocellular carcinoma (CC) (both ICD-9 code 155.0 and ICD-O-2 Morphology code 8180/3), and 4 (1.4%) were the mixed type of HCC and CC (both ICD-9 code 155.0 and ICD-O-2 Morphology code 8180/3).

One-to-one matched control subjects by sex and age (5-year interval) were selected from the “apparently healthy” people participating in a community-based diet and health survey in urban Guangzhou at that time. In that survey, the following conditions had already been excluded and further confirmed by the interview in this study: the abovementioned chronic diseases, extreme dietary intake, liver cirrhosis and cancer. A total of 1297 subjects recruited through community advertisements, written invitations, flyers and subjects’ referrals was screened. If more than one subject could be matched to one of the cases, the best-matched one was chosen for the controls. The final analysis included 644 case-patients and 644 matched control subjects (men 559 pairs, women 85 pairs). Written informed consent was obtained from each individual. The study was approved by the Ethics Committee of the School of Public Health at Sun Yat-sen University and was performed in accordance with the ethical standards laid down in the 1964 Declaration of Helsinki and its later amendments.

### Data collection

A personal interview was conducted by a trained research interviewer for each subject using a structured questionnaire. Except for hospital-documented data (e.g., clinical characteristics, clinical laboratory tests and instrument examinations) for PLC cases, the same questionnaires were used to collect the dietary data, general and health-related information, and other covariates mentioned below for both cases and controls in this study. Information on the socio-demographic characteristics (sex, age, education level, occupation, and household income), lifestyle behaviors (smoking, alcohol use, and drinking tea), physical activities, relevant diseases, and medications used over the previous 12 months was collected during the interview. The education levels were grouped into primary school or below, secondary school, and high school or above. Household income was divided into four levels (≤2000, 2001–4000, 4001–6000 and >6000 yuan/month/person). Occupations were grouped into light, moderate, and heavy activity levels according to the labor intensity. Smoking or alcohol use was defined as individuals who had smoked at least one cigarette/day or consumed any alcoholic beverages at least one time/week in six consecutive months. Individuals who drink tea at least twice a week were considered tea drinkers. Anthropometrics, including weight, height, and circumference at the waist and hips, were determined. The body mass index (BMI, kg/m^2^) was calculated.

### Dietary assessment

Dietary intake was ascertained using a quantitative 79-item Food Frequency Questionnaire (FFQ)^23^. Participants were asked to report the frequency (never, per year, per month, per week or per day) of intake and the portion size of their usually consumed foods over the past year before PLC was diagnosed for cases or interview for controls. We provided common food photographs of standard portion sizes to help the participants estimate the amount of food they had consumed. Daily food intake was calculated in grams per day. Total energy and individual nutrient content (folate, methionine and other related nutrients) were calculated based on the *China Food Composition Table 2004* ^29^. Daily choline and betaine intake were determined according to the food composition data analyzed by Zeisel *et al*. and the U.S. Department of Agriculture (USDA) Database^30, 31^. To evaluate choline intake, we calculated the contents of total choline and their components of free choline, phosphatidylcholine, phosphocholine, glycerophosphocholine and sphingomyelin. The energy-adjusted correlation coefficients for betaine and methionine ranged from 0.44 to 0.67 comparing the two FFQs six months apart and six 3-day dietary records^23^.

### Statistical analyses

All analyses were performed for men and women combined. The dietary intakes of choline, betaine and methionine were adjusted for total calories using the residual method^32^. The Wilcoxon signed-rank or McNemar’s test was used to test differences in the socio-demographic and nutrient intakes between the case and control subjects, as appropriate. Energy-adjusted choline, betaine and methionine intake were categorized into tertiles based on the distribution of these compounds in controls. We also calculated the total risk score by simply summing each score of the studied components. We assigned 0 or 1 point to each half of the participants according to the median (0 = <median, 1 = ≥median) for choline and betaine with linear association with PLC risk, or according to the quartiles (0 for extreme quartiles [Q1 and Q4], 1 for the middle quartile [Q2 and Q3]) of methionine with a U-shaped association. The subjects were then classified into four groups according to their values of the total risk score (0, 1, 2, and 3, higher point lower risk).

Conditional logistic regression was used to estimate the odds ratios (OR) and corresponding 95% confidence intervals (CIs) of PLC for the middle and high (*v*. low) tertiles for the intake of total choline, individual choline-containing compounds betaine, and methionine. In addition to matching by age and sex, we adjusted for the log-transformed energy intake (kcal/day) in Model 1, and we further adjusted for BMI, education (primary school or below, secondary school, high school or above), occupation (light, moderate or heavy activity), household income (≤1500, 1501–3000, 3000–6000, and >6000 yuan/month/person), currently smoking (yes or no), currently drinking (yes or no), tea drinking (yes or no) (a rich source of antioxidative polyphenols), physical activity (MET-h/day), and dietary folate intake (μg/d) in Model 2. The linear trends across increasing tertiles were tested by assigning tertile values as continuous variables in the regression analyses. We selected and considered the above covariates as potential confounders based on the theoretical relevance in the multivariable models.

We conducted stratified analyses and used multivariable unconditional logistic regression models to determine whether the aforementioned associations were modified by the folate intake (<309 [tertile 1] v. ≥309 [tertile 2 and 3] μg/d in controls), lifestyle behaviors (smoking [Yes/No], alcohol use [Yes/No], tea drinking [Yes/No]), BMI (<25 v. ≥25 kg/m2), age [≤54 v. >54 year], physical activity [≤33.3 v. >33.3 MET· h per day], education level [low/high] (low = secondary school or below; high = high school or above), HBV infection [Yes/No], diagnosis [Tissue evaluation/MRI imaging] and to calculate the multiplicative interactions by including each interaction item. All of the data were analyzed using SPSS, version 19.0. *P*-values were based on 2-sided tests and were considered significant at <0.05.

## Results

### Characteristics of the participants

Some selected characteristics of the 644 case-control pairs (559 male pairs and 85 female pairs) are shown in Table 1. The median age was 54.0 years in the PLC group and 54.0 years in the control group. PLC patients have lower BMI, physical activity, education level and a lower proportion of tea drinking, but there was a higher proportion of smokers, alcohol users, and workers with moderate or heavy labor jobs than that of the controls (all *P* < 0.05). HBV infection was found in 81.2% of the cases.

**Table 1:** Comparison of the general characteristics between primary liver cancer cases and controls^1^.

Characteristics	PLC (n = 644)	Controls (n = 644)	*P*
Age (years)	54.0 (46.0, 62.0)	54.0 (46.0, 63.0)	0.734^*^
Male, n (%)	559 (86.8)	559 (86.8)	1.000
BMI (kg/m^2^)	22.6 (20.7, 24.8)	23.1 (21.2, 25.2)	0.002^*^
Physical activity (MET· h per day)	28.9 (25.0, 36.4)	35.7 (32.1, 39.6)	<0.001^*^
Education level, n (%)			<0.001^*^
Primary school or below	155 (24.1)	74 (11.5)	
Secondary school	194 (30.1)	187 (29.0)	
High school or above	295 (45.8)	383 (59.5)	
Household income (yuan/month), n (%)			<0.004^*^
≤2,000	230 (35.7)	254 (39.4)	
2,000–4,000	239 (37.1)	249 (38.7)	
4,000–6,000	67 (10.4)	69 (10.7)	
>6,000	81 (12.6)	65 (10.1)	
Other	27 (4.2)	7 (1.1)	
Working strength, n (%)			0.001^*^
Light	246 (38.2)	343 (53.3)	
Moderate	155 (24.1)	86 (13.4)	
Heavy	177 (27.5)	158 (24.5)	
Other	66 (10.2)	57 (8.9)	
HBV infection, n (%)	523 (81.2)	not assessed	—
Smoking, n (%)	345 (53.6)	283 (43.9)	<0.001^#^
Alcohol user, n (%)	208 (32.3)	125(19.4)	<0.001^#^
Tea Drinker, n (%)	353 (54.8)	398 (61.8)	0.013^#^
Energy intake (kcal/d)^2^	1523 (1224, 1865)	1566 (1255, 1938)	0.004^*^
Total choline (mg/d)^3^	257 (208, 313)	288 (243, 343)	<0.001^*^
*Free choline* (*mg/d*)^*3*^	42 (33, 52)	50 (41, 61)	<0.001^*^
*Phosphatidylcholine* (*mg/d*)^*3*^	158 (122, 200)	178 (140, 220)	<0.001^*^
*Glycerophosphocholine* (*mg/d*)^*3*^	26 (21, 32)	28 (23, 34)	<0.001^*^
*Phosphocholine* (*mg/d*)^*3*^	11.0 (7.8, 15.9)	14.8 (11.0, 20.2)	<0.001^*^
*Sphingomyelin* (*mg/d*)^*3*^	13.0 (9.5, 16.9)	13.9 (11.0, 17.3)	0.001^*^
Betaine (mg/d)^3^	77 (48, 119)	93 (63, 130)	<0.001^*^
Methionine (mg/d)^3^	1957 (1713, 2288)	1867 (1676, 2068)	<0.001^*^
Folate (μg/d)^3^	339 (292, 398)	335 (297, 377)	0.497^*^

^1^Continuous values are medians (P25, P75).

^2^Energy intakes exclude cooking oil.

^3^Energy-adjusted intake.

^*^P values, Wilcoxon signed-rank test.

^#^P values, McNemar’s test.

Abbreviation: MET = metabolic equivalent.

### Dietary intake

The PLC patients consumed a slightly lower intake of total energy (1523 *v*. 1566, kcal/d, *P = *0.004) and had a lower energy-adjusted intake of betaine and total choline, but they had a higher intake of methionine than that of the controls (all *P* < 0.001). There was no significant difference in folate intake between the two groups. Of the total choline intake, phosphatidylcholine made the greatest contribution (61.8%), followed by free choline (17.4%), glycerophosphocholine (9.7%), phosphocholine (5.1%) and sphingomyelin (4.8%) in the controls (Table 1). The top five food sources for total choline were eggs (21.5%), streaky pork (13.0%), lean hogs (10.8%), Chinese flowering cabbage, mustard greens, collard greens and broccoli (8.7%) and poultry (4.0%). Grain products contributed to more than half of betaine intake (50.5%), followed by spinach. Rice, porridge and lean hogs were the main food sources of methionine (Supplemental Table S1).

### Association between choline, betaine and methionine intake and PLC risk

There were significant dose-dependent, favorable associations between the intake of choline and betaine with the PLC risk in both Models 1 and 2 (all *P*_*-*trend_ < 0.05). In Model 2, the ORs (95% CI) of PLC for the middle and high (vs. low) tertiles were 0.50 (0.36, 0.69) and 0.34 (0.24, 0.49) for total choline, and 0.67 (0.49, 0.92) and 0.67 (0.48, 0.93) for betaine. However, a significant association between the intake of methionine and PLC risk remained only in men (OR 1.71 [95% CI, 1.22, 2.39]) (Supplemental Table S3), but not in total subjects (OR 1.27 (95% CI, 0.93, 1.73)) in Model 2. (Table 2).

**Table 2:** Odds ratios (ORs) and 95% confidence intervals (CIs) of primary liver cancer according to the tertiles of total choline, betaine, methionine intake and total risk score.

	Amount (mg/d)	n (cases/controls)	Model 1	Model 2
			OR (95% CI)	OR (95% CI)
Tertile of choline				
T1	<258	327/214	1.00	1.00
T2	258–323	177/216	0.53 (0.40, 0.69)	0.50 (0.36, 0.69)
T3	>323	140/214	0.38 (0.28, 0.52)	0.34 (0.24, 0.49)
*P*-trend*			<0.001	<0.001
Tertile of betaine				
T1	<73	302/215	1.00	1.00
T2	73–116	172/215	0.56 (0.43, 0.74)	0.67 (0.49, 0.92)
T3	>116	170/214	0.56 (0.43, 0.74)	0.67 (0.48, 0.93)
*P*-trend			<0.001	0.011
Tertile of methionine				
T1	<1744	183/214	1.00	1.00
T2	1744–1989	162/215	0.88 (0.66, 1.17)	0.80 (0.57, 1.11)
T3	>1989	299/215	1.64 (1.25, 2.14)	1.27 (0.93, 1.73)
*P*-trend*			<0.001	0.104
Total Risk Score^#^				
0		132/59	1.00	1.00
1		261/193	0.60 (0.42, 0.86)	0.57 (0.38, 0.84)
2		191/260	0.33 (0.23, 0.47)	0.31 (0.21, 0.47)
3		60/132	0.20 (0.13, 0.31)	0.21 (0.13, 0.34)
*P*-trend*			<0.001	<0.001

Models 1 and 2: from the conditional logistic model.

Model 1: adjusted for the daily energy intake (ln-transformed), in addition to matching by sex and age.

Model 2: Model 1+, BMI, education level, occupation, household income, smoking, alcohol consumption, tea consumption, physical activity and dietary folate intake. The covariates were selected for their theoretical importance as confounders.

*α for p-trends = 0.05/6(tests) = 0.0083.

^#^Summing the scores of each nutrient of choline (0 = <median, 1 = ≥median), betaine (0 = <median, 1 = ≥median), and methionine (0 = quartile 1 and 4, 1 = quartile 2 and 3).

We further analyzed the association between individual choline compounds and PLC risk (Table 3). All of the individual choline compounds (free choline, glycerophosphocholine, phosphocholine, phosphatidylcholine, and sphingomyelin) were inversely associated with the risk of PLC (all P-trend < 0.01) in Model 2.

**Table 3:** Odds ratios (ORs) and 95% confidence intervals (CIs) of primary liver cancer according to the tertiles of the intake of five main choline-containing compounds in all subjects.

	Amount (mg/d)	n (cases/controls)	Model 1	Model 2
			OR (95% CI)	OR (95% CI)
Tertile of free choline				
T1	<44.1	356/214	1.00	1.00
T2	44.1–56.7	160/215	0.45 (0.33, 0.60)	0.47 (0.34, 0.66)
T3	>56.7	128/215	0.34 (0.25, 0.46)	0.36 (0.25, 0.51)
*P*-trend*			<0.001	<0.001
Tertile of glycerophosphocholine				
T1	<24.7	263/214	1.00	1.0
T2	24.7–31.5	217/215	0.82 (0.62, 1.07)	0.92 (0.67, 1.26)
T3	>31.5	164/215	0.62 (0.47, 0.81)	0.61 (0.44, 0.84)
*P*-trend			0.001	0.003
Tertile of phosphocholine				
T1	<12.4	373/214	1.00	1.00
T2	12.4–18.2	167/215	0.47 (0.35, 0.62)	0.45 (0.32, 0.62)
T3	>18.2	104/215	0.26 (0.19, 0.36)	0.26 (0.18, 0.38)
*P*-trend*			<0.001	<0.001
Tertile of phosphatidylcholine				
T1	<153.5	299/214	1.00	1.00
T2	153.5–206.0	194/216	0.66 (0.51, 0.86)	0.61 (0.44, 0.83)
T3	>206.0	151/214	0.47 (0.35, 0.63)	0.40 (0.28, 0.57)
*P*-trend*			<0.001	<0.001
Tertile of sphingomyelin				
T1	<12.2	284/214	1.00	1.00
T2	12.2–15.8	164/215	0.57 (0.44, 0.76)	0.58 (0.42, 0.81)
T3	>15.8	196/215	0.66 (0.51, 0.87)	0.56 (0.41, 0.77)
*P*-trend *			0.002	<0.001

Models 1 and 2: from the conditional logistic model.

Model 1: adjusted for the daily energy intake (ln-transformed), in addition to matching by sex and age.

Model 2: Model 1+, BMI, education level, occupation, household income, smoking, alcohol consumption, tea consumption, physical activity and dietary folate intake.

*α for *P*-trends = 0.05/10(tests) = 0.005.

### Total risk score and PLC risk

Both Model 1 and Model 2 showed a very significantly graded association between higher scores and a lower risk of PLC. The ORs (95% CI) of PLC for the categories of a total risk score of 0, 1, 2 and 3 were 1.00, 0.57 (0.38, 0.84), 0.31 (0.21, 0.47), and 0.21 (0.13, 0.34), respectively. The association tended to be more significant than those of each individual one-carbon-related nutrient (Table 2).

### Stratified and sensitivity analyses

We found that the favorable associations between the intake of betaine, total choline and PLC risk were not significantly different according to the subgroups of folate intakes (<309 *v* ≥309 μg/d) (*P*_-interaction_ = 0.238–0.618) (Table 4), gender, education, BMI, physical activity, smoking, alcohol drinking, and tea drinking (p-interactions: all >0.05/36 tests). However, the unfavorable association between higher methionine and PLC risk tended to be significant in older subjects (age: >54 vs. ≤54 years) (p-interaction < 0.001). Because there were no data of the HBV status and PLC tissue evaluation in the control group, we separately analyzed the relevant associations according to the PLC subjects’ status of HBV infectious (yes/no) and tissue evaluation (vs. MRI) using the same 644 control subjects. Similar trends were observed in the PLC subjects with/without HBV or using tissue evaluation. (Supplemental Table S2).

**Table 4:** Associations between the tertiles of total choline, betaine, methionine intake and primary liver cancer risk stratified by folate intake^1^.

	n (cases/controls)	OR (95% CI)	n (cases/controls)	OR (95% CI)
Folate intake^2^		<309 μg/d		≥309 μg/d
Tertile of total choline				
T1	104/71	1.00	214/143	1.00
T2	70/72	0.59 (0.35, 1.00)	123/143	0.52 (0.36, 0.74)
T3	45/72	0.35 (0.19, 0.62)	88/143	0.30 (0.20, 0.45)
*P*-trend*		<0.001		<0.001
*P*-interaction^#^	0.618			
Tertile of betaine				
T1	111/71	1.00	193/143	1.00
T2	53/72	0.59 (0.34, 1.02)	118/143	0.69 (0.49, 0.99)
T3	55/72	0.54 (0.31, 0.94)	114/143	0.62 (0.43, 0.88)
*P*-trend *		0.024		0.008
*P*-interaction^#^	0.237			
Tertile of methionine				
T1	85/71	1.00	103/143	1.00
T2	47/72	0.58 (0.32, 1.04)	96/143	0.95 (0.65, 1.40)
T3	87/72	1.16 (0.66, 2.04)	226/143	2.01 (1.44, 2.92)
*P*-trend*		0.518		<0.001
*P*-interaction^#^	0.383			
Total Risk Score				
0	48/24	1.00	84/35	1.00
1	100/80	0.65 (0.33, 1.25)	161/113	0.54 (0.33, 0.90)
2	63/88	0.39 (0.20, 0.78)	128/172	0.27 (0.16, 0.45)
3	8/23	0.13 (0.05, 0.39)	52/109	0.18 (0.10, 0.32)
*P*-trend*		<0.001		<0.001
*P*-interaction^#^	0.591			

ORs (95% CIs): from multivariate unconditional logistic regression models.

^1^Adjusted for the daily energy intake (ln-transformed), BMI, education level, occupation, household income, smoking, alcohol consumption, tea consumption, and physical activity.

^2^Stratified according to the lowest tertile intake of folate in the controls.

*α for *p*-trends = 0.05/6 (tests) = 0.0083.

^#^α for *p*-interactions = 0.05/3 (tests) = 0.0167.

## Discussion

In this case-control study, we found that a higher intake of total choline, as well as that of betaine, was associated with a reduced PLC risk. A high intake of methionine tended to be associated with a greater PLC risk. To the best of our knowledge, this was the first study to report the favorable association between choline and betaine consumption with PLC risk in humans. Our findings suggested that increasing the intakes of choline and betaine may be associated with a lower risk of PLC in humans.

Many studies have examined the associations between the consumption of dietary choline and betaine with cancer risk in animal models and humans. In the 1980s, it was reported that choline- and methionine-deficient diets could independently act as a complete carcinogen to develop hepatocellular carcinoma in rats without the administration of carcinogens^33, 34^. Some^13, 14, 17, 23, 35^ but not all^18, 20, 21, 25^ human studies have found a favorable association between dietary or circulating levels of choline and betaine and the risk of some cancers. A case-control study, nested within the European Prospective Investigation into Cancer and Nutrition (EPIC)^35^, including 1,367 colorectal cancer (CRC) cases and 2,323 controls, showed an inverse association between the plasma choline concentration and CRC risk in women (but not in men) and between the plasma betaine level and CRC risk in people with a lower plasma folate concentration^35^. Favorable associations between the dietary intakes of choline and betaine were also observed with respect to the risks of nasopharyngeal carcinoma (NPC)^23^, breast cancer^13^ and lung cancer^14^, although null^18, 20, 25^ or adverse^18, 21^ associations were also found in some other cancer studies. To date, only one case-control study has reported the relationship between the levels of choline or betaine in the diet or blood and the risk of hepatocellular carcinoma^27^. A recent meta-analysis showed that the pooled relative risks (95% CIs) of cancer for the highest versus lowest range of dietary intake were 0.82 (0.70 to 0.97) for choline, 0.86 (0.76 to 0.97) for betaine, and 0.60 (0.40 to 0.90) for choline and betaine combined^26^. Generally, consistent with the results of many other cancer studies, our findings support the hypothesis that a greater consumption of choline and betaine is associated with a lower risk of PLC in humans.

Regarding the individual components of the total dietary choline, phosphatidylcholine contributed more than 60% of the total choline, which was followed by free choline, glycerophosphocholine, phosphatidylcholine and sphingomyelin. We observed a significantly favorable association between consumption and PLC risk for these five components in our study. Favorable associations of these five components with total choline were also observed with respect to the risks of NPC, breast cancer and colorectal cancer in three different case-control studies that were conducted in the same geographic area^12, 13, 23^; although an inverse association with breast cancer risk was only observed for free choline intake in a cohort study^36^. Different choline components have different characteristics and varied food sources. Free choline, phosphocholine and glycerophosphocholine are water soluble and rich in cruciferous vegetables, whereas the other two types (phosphatidylcholine and sphingomyelin) are fat soluble and are mainly found in egg and animal foods (Supplemental Table 1). The strong internal consistency of the results for the different choline types that are present in different foods suggests that choline, rather than other co-existing food components, is most likely responsible for the favorable association between the total choline intake and PLC risk.

Biological explanations for the beneficial associations between high choline and betaine intake and PLC risk are still unclear. One potential mechanism that mediates the relationship between choline, betaine intake and primary liver cancer may be from their potential role in one-carbon metabolism. One-carbon metabolism can affect DNA methylation levels, particularly methylation at the CpG site, which plays an important role in genome regulation^37, 38^. The disruption of DNA methylation and impaired DNA repair induced by the deficiency of methyl donors (choline, betaine or methionine) in one-carbon metabolism was considered to contribute to the underlying mechanism of carcinogenesis^39^. Animals that are fed diets that lack choline and/or folic acid could induce profound changes in the expression of genes in the liver^40^ and development of hepatocellular carcinoma^41^. Next, choline, especially phosphatidylcholine, which is a predominant component of phospholipids in VLDL cholesterol, is necessary for TG transport from the liver, which may protect against liver fat accumulation^42^. On the other hand, some studies have suggested that high choline or betaine intake would improve the mitochondrial membrane and protect against oxidative stress and inflammation^43, 44^. Both fat accumulation and oxidative stress seem to be critical factors in the pathogenesis of chronic liver injury, including liver cancer.

Methionine, which is an indispensable amino acid, plays many key roles in mammalian metabolism, such as in protein synthesis, oxidative stress protection, DNA methylation and polyamine synthesis^45^. Conflicting results were observed between methionine and human cancers. In the Shanghai Women’s Health Study, subjects with a higher intake of methionine (>*vs*. <the median) had a 22% lower risk (HR: 0.78; 95% CI: 0.60–0.99) of lung cancer^16^. A prospective study comprising 81,922 individuals in a Swedish population with a mean follow-up of 7.2 years also found that a higher methionine intake could reduce the risk of pancreatic cancer^24^. Similar favorable associations between methionine intake and breast cancer were observed in a case-control study in women with positive estrogen receptor (ER+) status^15^. However, there were null associations between the dietary intake of methionine and cancer risk in the breast, endometrium, ovary, colorectal and lung in a large cohort of Canadian women who were followed-up for an average of 16.4 years^19^. Moreover, an adverse relationship between serum methionine and hepatocellular carcinoma risk was found in a nested case-control study in a cohort of 18,244 men in Shanghai^27^. In the present study, no significant relationship was observed between the dietary intake of methionine and PLC risk, and this result needs to be confirmed by more studies.

Folate and choline (through betaine) share methylation pathways (Supplemental Figure S1). Studies among animals and humans have shown that the folate and choline pathways may affect each other. Rats that were fed a folate-deficient diet showed depletion of the hepatic choline concentrations^46^. In the Framingham Offspring Study, higher intake levels of dietary choline and betaine were related to lower tHcy concentrations, which were limited to participants with low folate intake (<250 μg/d; *P*-_interaction_ < 0.0001)^47^. In this study, however, no significant interactions were observed between folate intake and the intake of choline, betaine and methionine. Further studies are needed to clarify whether choline and folate may exert complementary cancer prevention effects.

Some limitations of our study should be considered when interpreting the results. First, the case-control study design prevents conclusions concerning causality. We could not eliminate recall bias, i.e. knowledge of PLC diagnosis affecting dietary reporting by cases, or reverse causation, i.e. the disease affecting actual dietary intake, but we attempted to account for the design weaknesses in several ways. We enrolled only incident PLC cases, diagnosed within 1 month of survey. We used a food frequency questionnaire to assess dietary intake over the relatively long term (one year prior to PLC diagnosis), and we calculated energy-adjusted choline, betaine and methionine intakes to account for possible appetite changes due to PLC. Furthermore, recall bias is unlikely to be a problem, because the public was unfamiliar with the studied risk factors choline, betaine and methionine, which come from a large variety of “healthy” and “unhealthy” food sources. We also excluded people diagnosed with a major chronic disease within 5 years prior to survey, which probably minimized substantial dietary change. However, the cases and controls were thus healthier than the source population, which might lower the generalizability of our results. Our findings require further verification in a large sample of prospective cohort studies. Second, random recall error of habitual diets may be unavoidable in retrospective studies, although we used a large number of food photographs with standardized portion sizes to help participants estimate the amount of food they consumed to control for potential random errors and bias due to their recall ability. However, the potential bias was unlikely to be differential because the public was unfamiliar with the studied risk factors for choline, betaine and methionine with a large variety of different food sources, whereas non-differential recall bias tended to attenuate the association to null. Therefore, it is unlikely that we overestimated the favorable association in this study. Third, PLC patients were recruited from Sun Yat-Sen University Cancer Center, while the controls were recruited from local communities, which may influence the representativeness of the results. However, there were no significant differences in the studied associations between the pairs with the local cases and nonlocal cases (P-interactions between geographic area and choline/betaine: 0.332/0.838), suggesting the influence of different geographic regions would be limited. Fourth, case-control studies are prone to various selection biases. We reduced the prevalence-incidence bias by only including incident cases. Case admission and detection were unlikely to be related to the intake of choline, betaine, or methionine mainly consumed from usual foods. The studied PLC cases included in this study had a similar age (52 vs. 52* years, *this study), proportion of males (86% vs. 86.8%), and proportion with HBV infection (77% vs. 71.3%) but a relatively higher proportion of tobacco use (36% vs. 53.6%) and alcohol drinking (24% vs 32.3%), compared to a large HCC study containing 8,683 patients from 10 cities in China^48^ However, the studied associations were not significantly modified by BMI, education level, physical activity, smoking, alcohol drinking, tea drinking and HBV status, although we could not exclude the differences between the studied and target populations due to the convenient sampling. Fifth, we could not exclude the possible influence of HBV infection due to the lack of data in the controls. HBV infection was unlikely to be a significant confounder because no statistical association was found between the intake of choline, betaine and methionine and HBV infection among the cases (all P > 0.10). Sensitivity analysis showed that the studied associations were similar in patients with/without HBV infection. Finally, the limited study size did not allow us to obtain precise results in women.

In conclusion, our study suggested that higher intakes of dietary choline and betaine were inversely associated with primary liver cancer risk. Prospective studies are needed to confirm these findings.

## Electronic supplementary material

supplementary information
