# Supplementary material for: Higher dietary intakes of choline and betaine are associated with a lower risk of primary liver cancer: a case-control study
Source: Sci Rep. 2017 Apr 6;7:679. doi: 10.1038/s41598-017-00773-w (PMC5429604; doi:10.1038/s41598-017-00773-w)
Supplement: Supplementary file 1 — supplementary information [file 41598_2017_773_MOESM1_ESM.pdf]

Higher dietary intakes of choline and betaine are associated with a lower risk of primary liver cancer: a case-control study

Rui-fen Zhou[1#], Xiao-Lin Chen[1#], Zhong-guo Zhou[2], Yao-jun Zhang[2], Qiu-ye Lan[1], Gong-cheng Liao[1], Yu-ming Chen[1], Hui-lian Zhu[1*]

[1] School of Public Health, Sun Yat-sen University, Guangzhou (510080), People's Republic of China;

[2] Department of Hepatobiliary Surgery, Sun Yat-sen University Cancer Center, Guangzhou (510080), People's Republic of China

[#] These authors contributed equally to this work.

[*] The corresponding author:

Hui-lian Zhu, PhD, MD

Professor

Department of Nutrition

School of Public Health

Sun Yat-sen University

Guangzhou 510080

People's Republic of China

Email: zhuhl@mail.sysu.edu.cn

Tel.: +86 20 87331811

Fax: +86 20 87330446

Author List

Rui-fen Zhou:

Affiliations: Department of Nutrition , School of Public Health, Sun Yat-sen University, Guangzhou, P. R. China

E-mail address: 1123487506@qq.com

Work telephone number: +86 18824178816

Xiao-Lin Chen

Affiliations: Department of Nutrition , School of Public Health, Sun Yat-sen University, Guangzhou, P. R. China

E-mail address: 448037995@qq.com

Work telephone number: +86 3533184974

Zhong-guo Zhou:

Affiliations: Department of Hepatobiliary Surgery, Sun Yat-sen University Cancer Center, Guangzhou, P. R. China

E-mail address: zhouzhg@sysucc.org.cn

Yao-jun Zhang:

Affiliations: Department of Hepatobiliary Surgery, Sun Yat-sen University Cancer Center, Guangzhou, P. R. China

E-mail address: zhangyuj@sysucc.org.cn

Qiu-ye Lan:

Affiliations: Department of Nutrition , School of Public Health, Sun Yat-sen University, Guangzhou, P. R. China

E-mail address: bolivia.lan@gmail.com

Gong-cheng Liao:

Affiliations: Department of Nutrition , School of Public Health, Sun Yat-sen University, Guangzhou, P. R. China

E-mail address: 1368653532@qq.com

Yu-ming Chen:

Affiliations: Department of Medical Statistics & Epidemiology, School of Public Health, Sun Yat-sen University, Guangzhou, P. R.China

E-mail address: chenyum@mail.sysu.edu.cn

Hui-lian Zhu:

Affiliations: Department of Nutrition, School of Public Health,Sun Yat-sen University,Guangzhou, P. R. China

E-mail address: zhuhl@mail.sysu.edu.cn

Work telephone number : 0086 20 87331811

Work fax number: 0086 20 87330446

Supplemental Table S1. Top five contributors of food sources of total choline, main choline containing compounds and betaine among all subjects

Nutrients intake	Food source	Proportion (%)
Total choline (273.5 mg/d)	Eggs	21.5
	Streaky Pork	13.0
	Lean Hogs	10.8
	Chinese flowering cabbage, mustard greens, collard greens and broccoli	8.7
	Poultry	4.0
Phosphatidylcholine (167.9 mg/d)	Eggs	32.7
	Streaky Pork	16.1
	Lean Hogs	11.0
	Chinese flowering cabbage, mustard greens, collard greens and broccoli	7.3
	Freshwater fishes	4.4
Sphingomyelin (13.5 mg/d)	Streaky Pork	20.9
	Lean Hogs	18.7
	Eggs	18.1
	Poultry	14.2
	Regular front feet	6.2
Free choline (46.0 mg/d)	Chinese flowering cabbage, mustard greens, collard greens and broccoli	10.8
	Chinese cabbage, cauliflower, celery	8.1
	Chinese cabbage, leaf lettuce, and lettuce	7.6
	Rice	6.9
	Soymilk	5.1
Glycerophosphocholine (26.7 mg/d)	Lean Hogs	24.8
	Rice	15.3
	Streaky Pork	10.6
	Whole milk	9.9
	Porridge	5.7
Phosphocholine (13.1 mg/d)	Chinese flowering cabbage, mustard greens, collard greens and broccoli	40.5
	Chinese cabbage, leaf lettuce, and lettuce	6.5
	Whole milk	4.9
	Wax gourd, cucumber and zucchini	4.8
	Soymilk	4.7
Betaine (85.4 mg/d)	Noodles	26.1
	Spinach	23.4
	Steamed buns	14.9
	Whole-wheat bread	9.5
	Freshwater fishes	6.0

Methionine (1904 mg/d)	Rice	34.4
	Porridge	12.9
	Lean hogs	10.9
	Freshwater fishes	5.5
	Eggs	4.4

Supplemental Table S2. Associations between tertiles of total choline, betaine, methionine intake and primary liver cancer risk according to lifestyle behaviors[1]

	n (cases/controls)	OR (95% CI)	n (cases/controls)	OR (95% CI)
**Smoking**	**No**		**Yes**	
**Tertile of total choline**				
T1	161/120	1.00	155/94	1.00
T2	78/121	0.48 (0.32, 0.73)	104/95	0.64 (0.42, 0.97)
T3	60/120	0.38 (0.25, 0.59)	86/94	0.47 (0.30, 0.72)
P-trend		<0.001		0.001
P-interaction		0.406		
**Tertile of betaine**				
T1	133/120	1.00	171/94	1.00
T2	90/121	0.84 (0.56, 1.27)	79/95	0.48 (0.31, 0.73)
T3	76/120	0.76 (0.50, 1.16)	95/94	0.54 (0.35, 0.83)
P-trend		0.199		0.003
P-interaction		0.259		
**Tertile of methionine**				
T1	81/120	1.00	99/94	1.00
T2	68/121	0.77 (0.49, 1.20)	96/95	1.01 (0.65, 1.55)
T3	150/120	1.65 (1.10, 2.47)	150/94	1.25 (0.83, 1.90)
P-trend		0.008		0.276
P-interaction		0.218		
**Score ***				
0	62/27	1.00	70/32	1.00
1	116/112	0.39 (0.22, 0.71)	145/81	0.72 (0.42, 1.24)
2	86/136	0.26 (0.14, 0.47)	105/124	0.34 (0.20, 0.59)
3	35/86	0.19 (0.10, 0.38)	25/46	0.23 (0.11, 0.48)
P-trend		<0.001		<0.001
P-interaction		0.790		
**Alcohol use**	**No**		**Yes**	
**Tertile of total choline**				
T1	210/173	1.00	127/41	1.00
T2	132/173	0.63 (0.45, 0.87)	42/42	0.34 (0.18, 0.62)
T3	94/173	0.45 (0.32, 0.65)	39/42	0.31 (0.17, 0.57)
P-trend		<0.001		<0.001
P-interaction		0.070		
**Tertile of betaine**				
T1	207/173	1.00	94/41	1.00
T2	114/173	0.62 (0.44, 0.86)	70/42	0.82 (0.46, 1.47)
T3	115/173	0.61 (0.44, 0.87)	44/42	0.55 (0.30, 1.03)
P-trend		0.004		0.064
P-interaction		0.918		

**Tertile of methionine**				
T1	117/173	1.00	68/41	1.00
T2	108/173	0.93 (0.65, 1.32)	61/42	0.91 (0.49, 1.68)
T3	211/173	1.73 (1.24, 2.42)	79/42	1.04 (0.58, 1.86)
P-trend		0.001		0.894
P-interaction			0.179	
**Score**				
0	93/45	1.00	39/14	1.00
1	176/167	0.44 (0.28, 0.69)	85/26	1.12 (0.50, 2.49)
2	127/210	0.26 (0.16, 0.41)	64/50	0.48 (0.22, 1.04)
3	40/97	0.20 (0.11, 0.36)	20/35	0.26 (0.10, 0.63)
P-trend		<0.001		<0.001
P-interaction			0.669	
**Drink tea**		**No**		**Yes**
**Tertile of total choline**				
T1	139/82	1.00	176/132	1.00
T2	80/82	0.56 (0.36, 0.89)	115/133	0.62 (0.43, 0.90)
T3	72/82	0.42 (0.26, 0.67)	62/133	0.35 (0.23, 0.54)
P-trend		<0.001		<0.001
P-interaction			0.360	
**Tertile of betaine**				
T1	115/82	1.00	167/132	1.00
T2	97/82	0.88 (0.56, 1.37)	97/133	0.80 (0.54, 1.17)
T3	79/82	0.64 (0.41, 1.02)	89/133	0.66 (0.44, 0.98)
P-trend		0.062		0.038
P-interaction			0.399	
**Tertile of methionine**				
T1	76/82	1.00	106/132	1.00
T2	71/82	0.96 (0.60, 1.55)	90/133	0.75 (0.50, 1.13)
T3	144/82	1.96 (1.25, 3.06)	157/133	1.16 (0.79, 1.71)
P-trend		0.002		0.385
P-interaction			0.401	
**Score**				
0	63/28	1.00	69/31	1.00
1	127/91	0.52 (0.29, 0.92)	134/102	0.57 (0.33, 0.99)
2	79/92	0.31 (0.17, 0.57)	112/168	0.30 (0.17, 0.51)
3	22/35	0.18 (0.08, 0.40)	38/97	0.23 (0.12, 0.43)
P-trend		<0.001		<0.001
P-interaction			0.686	
**BMI(kg/m2)**		**<25**		**⩾25**
**Tertile of total choline**				
T1		1.00		1.00
T2	252/157	0.58 (0.41, 0.80)	69/57	0.51 (0.28, 0.91)

T3	141/157	0.39 (0.27, 0.56)	43/58	0.37 (0.20, 0.70)
P-trend	107/157	<0.001	32/58	0.002
P-interaction		0.929		
**Tertile of betaine**				
T1	247/157	1.00	54/57	1.00
T2	131/157	0.61 (0.44, 0.85)	46/58	0.94 (0.52, 1.71)
T3	122/157	0.51 (0.36, 0.73)	44/58	1.03 (0.57, 1.88)
P-trend		<0.001		0.920
P-interaction		0.044		
**Tertile of methionine**				
T1	150/157	1.00	40/57	1.00
T2	119/157	0.82 (0.58, 1.17)	27/58	0.77 (0.40, 1.48)
T3	231/157	1.47 (1.06, 2.03)	77/58	1.80 (1.00, 3.23)
P-trend		0.016		0.034
P-interaction		0.547		
**Score**				
0	106/41	1.00	26/18	1.00
1	205/140	0.52 (0.33, 0.81)	56/53	0.73 (0.33, 1.63)
2	145/195	0.27 (0.17, 0.43)	46/65	0.49 (0.22, 1.08)
3	44/95	0.19 (0.11, 0.33)	16/37	0.35 (0.14, 0.88)
P-trend		<0.001		0.009
P-interaction		0.228		
**Age (years)**		**≤54**		**>54**
**Tertile of total choline**				
T1	161/108	1.00	166/106	1.00
T2	98/109	0.55 (0.37, 0.82)	79/107	0.53 (0.34, 0.82)
T3	66/108	0.31 (0.20, 0.49)	74/106	0.51 (0.33, 0.80)
P-trend		<0.001		0.002
P-interaction		0.486		
**Tertile of betaine**				
T1	119/108	1.00	181/106	1.00
T2	101/109	0.82 (0.55, 1.24)	64/107	0.46 (0.29, 0.71)
T3	105/108	0.88 (0.58, 1.32)	74/106	0.49 (0.32, 0.76)
P-trend		0.541		0.001
P-interaction		0.019		
**Tertile of methionine**				
T1	124/108	1.00	65/106	1.00
T2	76/109	0.61 (0.40, 0.92)	69/107	0.95 (0.58, 1.55)
T3	125/108	0.93 (0.63, 1.37)	185/106	2.74 (1.75, 4.28)
P-trend		0.707		<0.001
P-interaction		<0.001		
**Score**				
0	68/40	1.00	64/19	1.00

1	136/109	0.71 (0.43, 1.17)	125/84	0.39 (0.20, 0.75)
2	95/131	0.39 (0.23, 0.65)	96/129	0.22 (0.11, 0.42)
3	26/45	0.29 (0.15, 0.57)	34/87	0.15 (0.07, 0.31)
P-trend		<0.001		<0.001
P-interaction			0.155	
**Physical activity (MET· h per day)**		**≤33.3**		**>33.3**
**Tertile of total choline**				
T1	210/71	1.00	125/143	1.00
T2	127/72	0.45 (0.27, 0.75)	57/143	0.50 (0.32, 0.77)
T3	92/72	0.42 (0.24, 0.73)	33/143	0.27 (0.16, 0.44)
P-trend		0.001		<0.001
P-interaction			0.047	
**Tertile of betaine**				
T1	192/71	1.00	112/143	1.00
T2	122/72	0.94 (0.56, 1.56)	55/143	0.61 (0.40, 0.95)
T3	115/72	0.88 (0.52, 1.51)	48/143	0.51 (0.32, 0.80)
P-trend		0.643		0.003
P-interaction			0.225	
**Tertile of methionine**				
T1	109/71	1.00	69/143	1.00
T2	102/72	0.93 (0.54, 1.62)	53/143	0.79 (0.50, 1.25)
T3	218/72	1.52 (0.90, 2.54)	93/143	1.28 (0.84, 1.97)
P-trend		0.091		0.225
P-interaction			0.245	
**Score**				
0	81/11	1.00	51/48	1.00
1	167/67	0.31 (0.14, 0.70)	94/126	0.76 (0.45, 1.27)
2	138/91	0.22 (0.10, 0.51)	53/169	0.36 (0.21, 0.62)
3	43/46	0.16 (0.07, 0.41)	17/86	0.25 (0.12, 0.51)
P-trend		<0.001		<0.001
P-interaction			0.831	
**Education level**		**Low**		**High**
**Tertile of total choline**				
T1	178/87	1.00	138/127	1.00
T2	105/87	0.54 (0.35, 0.83)	87/128	0.53 (0.35, 0.81)
T3	66/87	0.31 (0.20, 0.49)	70/128	0.42 (0.27, 0.66)
P-trend		<0.001		<0.001
P-interaction			0.327	
**Tertile of betaine**				
T1	161/87	1.00	130/127	1.00
T2	96/87	0.65 (0.43, 1.01)	81/128	0.75 (0.49, 1.13)
T3	92/87	0.56 (0.36, 0.86)	84/128	0.78 (0.51, 1.18)

P-trend		0.008		0.216
P-interaction		0.359		
**Tertile of methionine**				
T1	116/87	1.00	74/127	1.00
T2	80/87	0.64 (0.41, 0.99)	73/128	0.98 (0.62, 1.53)
T3	153/87	1.16 (0.77, 1.76)	148/128	1.79 (1.17, 2.72)
P-trend		0.429		0.004
P-interaction		0.139		
**Score**				
0	84/32	1.00	48/27	1.00
1	157/101	0.55 (0.33, 0.93)	104/92	0.64 (0.34, 1.20)
2	89/100	0.31 (0.18, 0.53)	102/160	0.38 (0.20, 0.69)
3	19/28	0.21 (0.09, 0.46)	41/104	0.25 (0.13, 0.50)
P-trend		<0.001		<0.001
P-interaction		0.702		
**HBV infectious**	**No**		**Yes**	
**Tertile of total choline**				
T1	61/214	1.00	266/214	1.00
T2	29/215	0.47 (0.27, 0.80)	148/215	0.53 (0.39, 0.72)
T3	31/215	0.37 (0.21, 0.64)	109/215	0.37 (0.27, 0.52)
P-trend		<0.001		<0.001
**Tertile of betaine**				
T1	63/214	1.00	239/214	1.00
T2	32/215	0.55 (0.33, 0.92)	141/215	0.68 (0.50, 0.92)
T3	26/215	0.42 (0.24, 0.73)	143/215	0.66 (0.48, 0.89)
P-trend		0.002		0.006
**Tertile of methionine**				
T1	35/214	1.00	148/214	1.00
T2	26/215	0.72 (0.40, 1.30)	136/215	0.91 (0.66, 1.25)
T3	60/215	1.43 (0.86, 2.38)	239/215	1.55 (1.14, 2.09)
P-trend		0.128		0.003
**Score**				
0	29/59	1.00	103/59	1.00
1	48/193	0.48 (0.26, 0.90)	213/193	0.59 (0.39, 0.89)
2	33/260	0.23 (0.12, 0.45)	158/260	0.33 (0.22, 0.49)
3	11/132	0.17 (0.07, 0.40)	49/132	0.22 (0.13, 0.36)
P-trend		<0.001		<0.001
**Diagnosis**	**Tissue evaluation**		**MRI imaging**	
**Tertile of total choline**				
T1	124/214	1.00	203/214	1.00
T2	57/215	0.43 (0.28, 0.64)	120/215	0.60 (0.43, 0.83)
T3	46/215	0.33 (0.21, 0.51)	94/215	0.41 (0.29, 0.58)
P-trend		<0.001		<0.001

**Tertile of betaine**				
T1	112/214	1.00	190/214	1.00
T2	69/215	0.71 (0.48, 1.04)	104/215	0.63 (0.45, 0.88)
T3	46/215	0.46 (0.30, 0.70)	123/215	0.71 (0.51, 0.99)
P-trend		<0.001		0.032
**Tertile of methionine**				
T1	62/214	1.00	121/214	1.00
T2	72/215	1.15 (0.76, 1.74)	90/215	0.72 (0.50, 1.02)
T3	93/215	1.36 (0.91, 2.04)	206/215	1.54 (1.12, 2.13)
P-trend		0.135		0.004
**Score**				
0	47/59	1.00	85/59	1.00
1	96/193	0.54 (0.33, 0.91)	165/193	0.55 (0.36, 0.85)
2	65/260	0.28 (0.16, 0.47)	126/260	0.32 (0.21, 0.49)
3	19/132	0.17 (0.09, 0.34)	41/132	0.22 (0.13, 0.38)
P-trend		<0.001		<0.001

ORs (95% CI): from multivariate unconditional logistic regression models.

[1]adjusted for sex, age, daily energy intake (ln-transformed), BMI, education level, occupation, household income, smoking, alcohol consumption, drink tea, physical activity.

P-trend: α for p-trends = 0.05/6(tests) = 0.0083

Abbreviation: CI=confidence interval

*: summing the scores of each nutrient of choline (0= < median, 1= ≥ median), betaine (0= < median, 1= ≥ median), and methionine (0 = quartile 1 and 4, 1= quartile 2 and 3)

Supplemental Table S 3 . Odds ratios (OR) and 95% confidence intervals (CI) of primary liver cancer according to tertiles of total choline, betaine, methionine intake in men

	**Amount (mg/d)**	**n (cases/controls)**	**Model1 OR (95% CI)**	**Model2 OR (95% CI)**
**Tertile of choline**				
T1	<253	269/186	1.00	1.00
T2	253-315	161/186	0.61 (0.46, 0.81)	0.59 (0.44, 0.80)
T3	>315	129/187	0.48 (0.36, 0.64)	0.44 (0.32, 0.62)
P-trend			<0.001	<0.001
**Tertile of betaine**				
T1	<72	259/186	1.00	1.00
T2	72-117	149/186	0.57 (0.42, 0.75)	0.64 (0.47, 0.87)
T3	>117	151/187	0.57 (0.43, 0.76)	0.64 (0.47, 0.88)
P-trend			<0.001	0.005
**Tertile of methionine**				
T1	<1757	169/186	1.00	1.00
T2	1757-2004	136/186	0.81 (0.60, 1.10)	0.92 (0.66, 1.28)

T3	>2004	254/187	1.51 (1.14, 2.00)	1.71 (1.22, 2.39)
P-trend			0.002	0.001
**Tertile of folate（μg/d）**				
T1	<309	199/186	1.00	1.00
T2	309-360	160/186	0.81 (0.60, 1.09)	1.31 (0.91, 1.89)
T3	>360	200/187	1.02 (0.76, 1.35)	2.66 (1.57, 4.51)
P-trend			0.915	<0.001

Models 1 and 2: from conditional logistic model.

Model 1: adjusted for daily energy intake (ln-transformed), besides matched by sex and age.

Model 2: Model 1 +, BMI, education level, occupation, household income, smoking, drinking, drink tea, physical activity and dietary folate intake.
